# Population differentiation of the African cyprinid *Barbus neumayeri* across dissolved oxygen regimes

**DOI:** 10.1002/ece3.561

**Published:** 2013-04-17

**Authors:** Robert Harniman, Thomas J S Merritt, Lauren J Chapman, David Lesbarrères, Mery L Martinez

**Affiliations:** 1 Department of Biology, Laurentian University935 Ramsey Lake Road, Sudbury, Ontario, P3E 2C6, Canada; 2 Department of Chemistry and Biochemistry, Laurentian University935 Ramsey Lake Road, Sudbury, Ontario, P3E 2C6, Canada; 3 Department of Biology, McGill University1205 Ave. Dr Penfield, Montréal, Québec, H3A 1B1, Canada

**Keywords:** Cyprinidae, divergent selection, genetic structure, *G*_ST_, hypoxia, isolation by distance, Jost's *D*

## Abstract

Population level response to hypoxia has become an issue of global significance because of increased frequency and intensity of hypoxic events worldwide, and the potential for global warming to exacerbate hypoxic stress. In this study, we sequenced two nuclear intronic regions and a single mitochondrial region across seven populations of the African cyprinid, *Barbus neumayeri* from two river drainages in Uganda: the Rwembaita Swamp-Njuguta River System and the Dura River. We then examined two indices of population structure, *G*_ST_ and Jost's *D*, to detect links between oxygen availability and genetic variation and to determine if population genetic structure was associated with (i) dissolved oxygen regime (hypoxia or normoxia), (ii) geographical distance, or (iii) a combination of dissolved oxygen regime and geographical distance. Our results indicate that over a large scale (between drainages), geographical distance significantly affects the genetic structure of populations. However, within a single drainage, dissolved oxygen regime plays a key role in determining the genetic structure of populations. Within the Rwembaita-Njuguta system, gene flow was high between locations of similar oxygen regimes, but low between areas characterized by divergent oxygen regimes. Interestingly, *G*_ST_ analyses appear to yield less realistic measures of population structure than Jost's *D*, suggesting that caution must be taken when interpreting and comparing the results from different studies. These results support the idea that aquatic dissolved oxygen can act as a selective force limiting gene flow among populations of aquatic species and therefore should be considered when implementing conservation plans and assessing environmental impact of human activities.

## Introduction

Both geographical isolation and environmental differences can decrease population connectivity and contribute to genetic divergence between populations, particularly in fragmented landscapes (Hedrick 2000; Manel et al. 2003; Richardson 2012). Environmental factors can create barriers to migration and gene flow, potentially fixing life history changes, and exacerbating population fragmentation and isolation caused by distance. The role of environmental factors in genetic isolation, and the possibility that these factors may trump, exacerbate, or confound divergence resulting from geographical separation, has received renewed interest with environmental factors changing in response to the rise of global temperatures, and the secondary effects associated with global warming such as oxygen limitation of thermal tolerance (Davis and Shaw 2001; Gienapp et al. 2008; Pörtner 2010). For example, Qian and Davies (1996) showed that interpopulation genetic differences between populations of freshwater leeches (*Nephelopsis obscura*) residing in lakes with different dissolved oxygen (DO) regimes, were more consistent with oxygen acting as a selective agent than geographical distance. Similarly, at least two populations of the common skate (*Dipturus batis*) have been found to be reproductively isolated around the British Isles (Griffiths et al. 2010). Although both skate populations had the same depth and substrate preferences, reproduction and dispersal between the groups were limited due to areas of low water temperature, suggesting that differences in environmental temperature can cause genetic isolation of populations in an otherwise continuous environment (Griffiths et al. 2010). These are not unique examples, and several other systems also suggest that environmental differences can cause genetic isolation among populations (Reusch et al. 2001; Lecomte and Dodson 2004; Bekkevold et al. 2005; Funk et al. 2005; McCairns and Bernatchez 2008).

Several recent studies have demonstrated strong patterns of interdemic variation in fish populations between low- and high-oxygen environments, particularly in traits related to oxygen uptake such as gill size (Chapman et al. 1999; Timmerman and Chapman 2004; Chapman 2007; Binning et al. 2010; Tobler et al. 2011), and structural elements surrounding the gills (Chapman et al. 2008), but also brain size (Chapman and Hulen 2001; Chapman et al. 2008), and body shape (Langerhans et al. 2007; Crispo and Chapman 2011). These studies suggest that alternative dissolved oxygen regimes may represent a strong source of divergent natural selection that may lead to reduced gene flow and, potentially, ecological speciation. However, the degree to which genetic differences underlie the phenotypic divergence across dissolved oxygen regimes appears to vary across species. In their studies of the African cichlid *Pseudocrenilabrus multicolor victoriae*, Crispo and Chapman (2008) examined several populations from the Nabugabo and Mpanga River drainages of Uganda to investigate the potential for reduced gene flow between populations divergent in behavioral, morphological, and physiological traits. While the authors were able to detect genetic differences as a function of geographical distance using one mitochondrial and 12 microsatellite markers, no correlation between genetic divergence and the oxygen regime (hypoxic vs. normoxic) was observed, suggesting that phenotypic plasticity may be facilitating migration between ecologically different habitats in this system. Common garden rearing experiments in this same cichlid species showed evidence for very high levels of plasticity in gill size, and both plastic and genetics effects in brain mass (Crispo and Chapman 2010). A second widespread African fish, the cyprinid *Barbus neumayeri* that inhabits the higher order tributaries and upstream sectors of the Mpanga River drainage displays similar differences in behavioral, morphological, and biochemical traits between high- and low-dissolved oxygen sites (Chapman and Liem 1995; Olowo and Chapman 1996; Schaack and Chapman 2003; Martínez et al. 2004). However, in contrast to the cichlid example, a combination of long-term acclimation and allozyme studies suggest the possibility of genetic differentiation between oxygen regimes over small spatial scales in this species (Chapman et al. 1999; Martínez et al. 2004). In this study, we used nuclear introns (noted to be variable between fish populations, Quattro and Jones 1999; Quattro et al. 2001) to examine whether populations of *B. neumayeri* are genetically structured by (i) dissolved oxygen regime (hypoxia or normoxia), (ii) physical barriers such geographical distance, and/or (iii) both oxygen regime and geography. Nuclear introns accumulate change at a relatively fast rate (e.g., compared to coding regions) and can often be easily amplified from a species of interest using primers designed from the relatively conserved flanking coding regions.

Valley papyrus swamps in East and Central Africa provide an excellent system for exploring the role of dissolved oxygen as a divergent selective agent, because the dense interior of the papyrus swamps is hypoxic through the year (Chapman et al. 1999; Chapman and Hulen 2001), and these swamps represent patches of hypoxic habit in the matrix of the larger, flowing, and generally well-oxygenated rivers. In papyrus swamps, conditions of low light incidence caused by the height of papyrus plants (often up to 5 m) and low water mixture, in combination with the high amount of organic decomposition of papyrus and other plants, result in DO levels well below normoxic conditions (Chapman and Liem 1995; Chapman et al. 1999). Valley papyrus swamps can significantly reduce river valleys DO levels for several kilometres, potentially creating a barrier to hypoxia tolerant species. We use the largest papyrus swamp-river system in Kibale National Park, Uganda (Rwembaita Swamp), as the focus of our study because *B. neumayeri* is widely distributed in the park occurring in the dense interior of the papyrus as well as small tributary streams and the open waters of the larger rivers (Langerhans et al. 2007). Kibale National Park, a mid-altitude moist forest in equatorial Uganda, is drained by two major river systems (Dura and Mpanga) and associated swamp and stream tributaries that produce the mosaic of dissolved oxygen conditions in which *B. neumayeri* is very abundant. The substantial variation in DO between and among ecosystems in Kibale National Park represents a system of multiple, semi-isolated, environmental regions in which divergent natural selection may be selecting for individuals tolerant to a specific dissolved oxygen regime.

*Barbus neumayeri* exhibits strong patterns of phenotypic variation between low- and high-dissolved oxygen sites within the rivers of Kibale. Fish from hypoxic swamp sites are characterized by larger gills (Chapman et al. 1999; Langerhans et al. 2007), lower thresholds for aquatic surface respiration (Olowo and Chapman 1996), and lower critical oxygen tension (Chapman 2007) than conspecifics from nearby well-oxygenated stream and river sites. Moreover, individuals from hypoxic areas present a higher glycolytic capacity than fish from normoxic areas, even after acclimation and acclimatization periods (Martínez et al. 2004, 2011). The nonplastic differences in enzyme activity in fish from environments with different dissolved oxygen levels suggest that these differences are genetically based and that the populations of *B. neumayeri* residing in divergent oxygen regimes are genetically isolated. To test this potential isolation, we quantified genetic structure using two different methods, *G*_ST_ and Jost's *D*. *G*_ST_ the more established metric, quantifies the standardized variance in allele frequencies among populations. Jost's *D* coefficient, a more recent derived metric, is a measure of genetic differentiation (Jost 2008; Raeymaekers et al. 2012). Some recent work suggests that *G*_ST_ may under-estimate genetic differences and that in some cases, Jost's *D* will give a more accurate picture of the true genetic structure of populations (Jost 2008; Whitlock 2011). Here, we used both measures to test the genetic differentiation between *B. neumayeri* subpopulations sampled in the Mpanga and Dura River regions.

## Methods

### Study location description

**Figure 1:**
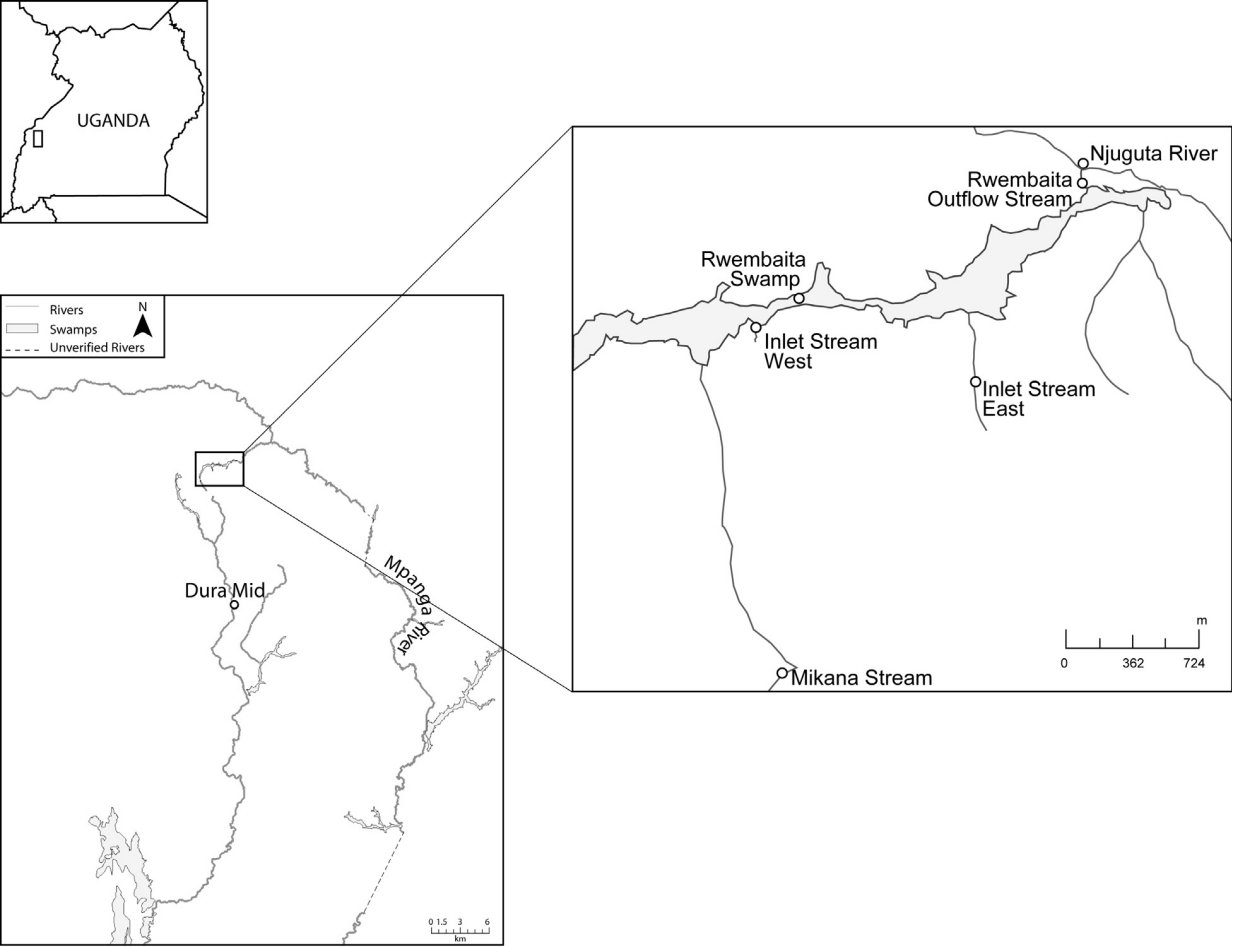
Map of the Dura and Mpanga drainages of the Lake George region, Kibale National Park, Uganda. Inset in the map of Uganda indicates the location of the sampling regions.

In this study, we sampled fish from seven locations in the Kibale National Park in Uganda, Africa. Sites were selected to maximize variation in dissolved oxygen (DO) and geographical distance. The two main rivers Dura River and the Mpanga River, both eventually feed into the Lake George system (Fig. 1). Basic physico-chemical characteristics of these sites including water current, water temperature, dissolved oxygen concentrations, maximum depth at capture locations, conductivity, pH, and water transparency are summarized in Langerhans et al. (2007). Six locations were within the Rwembaita Swamp-Njuguta River system (which feeds into the Mpanga River), and one location was located on the Dura River, deep within the forest. The Dura River location is located more than 100 km away on a separate tributary of Lake George, and is geographically located south of all of the other locations in the Rwembaita Swamp-Njuguta River system (Fig. 1). To disperse from the Dura River to the Mpanga River, fish would have to move downstream through a major papyrus swamp, and upstream through several kilometres of fast-flowing river, and transverse the Mpanga Falls (approx. 50 m in height). Therefore, contemporary gene flow between the Dura River location and the Rwembaita/Njuguta River locations is very unlikely, and a high amount of divergence between those locations was expected. The seven locations were characterized by divergent DO levels, varying from 1.35 mg O_2_ L^−1^ to 7.32 mg O_2_ L^−1^ (Langerhans et al. 2007). Selected locations included two swamp water locations, two river locations, and three stream locations (Fig. 1). The most western location in the Rwembaita-Njuguta system is the Mikana Stream, which flows eastward into the Rwembaita Swamp. Two additional small tributary streams that feed into the Rwembaita Swamp were sampled: Inlet Stream West with Inlet Stream East. A tributary of swamp water leaves the Rwembaita Swamp (referred to as Rwembaita/Njuguta Outflow Stream), and passes through secondary forest for 80 m before meeting the waters of the Njuguta River, the latter drains into the Mpanga River. DO levels in the Rwembaita Swamp have been recorded since the early 1990s, and DO has remained remarkably low over the years. Seasonal variation is apparent in the swamp water with higher DO during the rainy periods; however, the swamp water remains well below saturation throughout the year (Chapman and Liem 1995; Chapman et al., 1999; Langerhans et al. 2007; Joyner-Matos and Chapman, 2013).

### Fish sampling and DNA extraction

Fish were sampled during the rainy season in January 2010 using baited minnow traps, and body mass and total length were measured in a small plastic bag with water. Pectoral fin clips were taken and stored in 95% ethanol for DNA analyses. Once the fin clips were sampled, fish were allowed to recover in a bucket of well-oxygenated water from their habitat, and they were released at the location of capture. Genomic DNA was extracted using the DNeasy Tissue Kit (QIAGEN, Mississauga, ON, Canada), and DNA concentration was estimated using an ND-8000 NanoDrop spectrophotometer (ThermoScientific, Ottawa, ON, Canada).

### DNA sequence data

We used Polymerase chain reaction (PCR) to amplify two nuclear and one mitochondrial gene regions. The nuclear and mitochondrial loci have demonstrated utility in resolving question of population structure in other studies (e.g., Quattro and Jones 1999; Quattro et al. 2001). The nuclear loci are introns and although linkage to coding and/or regulatory variation is possible, they are likely to be neutral and any segregation of polymorphisms should reflect isolation of the populations not selection on these loci. Each 25 μL PCR reaction contained approximately 0.06 ng of template DNA, 2.5 μL of TopTaq Master Mix (QIAGEN), 2.5 μL CoralLoad (QIAGEN), and 1.25 μM, each, of specific forward and reverse primers. Reaction parameters were optimized for each primer set and are shown below. Reactions were checked for single amplification products (bands) using gel electrophoresis and ethidium bromide staining and sequenced at the Génome Québec Innovation Center using an Applied Biosystems 3730xl sequencer (Burlington, ON, Canada).

#### Creatine kinase

The sixth intron in the Creatine Kinase-A (CK-A) nuclear locus was amplified using an oligonucleotide primer set previously optimized for various fish species, including cyprinids (Quattro and Jones 1999). The primers are as follows: CKA7F1: 5′-CCC AAG TTY GAG GAG ATC CTG AC-3′ and CKA7R1: 5′-CCG TCG ACG ACC AGC TGC ACC TG-3′. These primers were designed to specifically amplify the 6th intron from the CK-A locus, and not the CK-B locus, to eliminate the possibility of inadvertently comparing paralogous sequences (Quattro and Jones 1999; Quattro et al. 2001). PCR conditions were: an initial denaturation of 2 min at 94°C followed by 35 cycles of 1 min at 94°C, 40 sec at 48°C, 2 min at 72°C, and a final extension time of 7 min at 72°C.

#### Triosephosphate isomerase

The fourth intron of the Triosephosphate Isomeras A (TPI-A) nuclear locus was amplified using locus specific primers designed in a similar fashion to the CK primers (J. M. Quattro pers. comm.). Amplification of sufficient product to allow direct sequencing required semi-nested primary and secondary PCR reactions. The primer sequences are as follows: TPIDF1: 5′-YTG ATY GGS CAG AAG GTG GC-3′; 302 – TPIB4 R1: 5′-AGA ACC ACY TTR CTC CAG TC-3′; TPI4F2 – P301: 5′-GCA TYG GGG AGA AGC TRG AT-3′. The primary amplification was from TPIDF1 to 302 – TPIB4 R1. From this reaction, 2 μL of product was diluted in 100 μL water and 2 μL of this solution was used as template in a second PCR reaction from TPI4F2 – P301 to 302 – TPIB4 R1. In both primary and secondary reactions, the PCR parameters were as follows: an initial denaturation of 2 min at 90°C followed by 44 cycles of 45 sec at 90°C, 60 sec at 48°C, 60 sec at 72°C and a final extension time of 7 min at 72°C.

#### 16S mitochondrial locus

A fragment of the 16S mitochondrial locus was amplified using “universal” primers: 16S AR: 5′-CGC CTG TTT AAC AAA AAC AT-3′; 16S BR: 5′-CCG GTC TGA ACT CAG ATC ACG T-3′ (Simon et al. 1994). The PCR protocol was: an initial denaturation of 2 min at 90°C followed by 35 cycles of 1 min at 90°C, 1 min at 48°C, 2 min at 72°C, and a final extension time of 7 min at 72°C.

### Data analysis

Raw sequence curve files were edited manually and aligned using CodonCode Aligner v 3.03 (CodonCode Corporation, Dedham, MA) with a final visual optimization of the alignment. The number of haplotypes (within and between location), haplotype diversity (*Hd*), nucleotide diversity (π), *G*_ST_ and *F*_ST-_values were all calculated using DnaSP version 4.10 (Rozas 2009). Haplotype frequencies among all populations and distance matrices were calculated using ARLEQUIN version 3.11 (Excoffier et al. 2005). In order to determine allelic similarity and dissimilarity within and between populations, we used the Jost's *D* coefficient, as defined by Jost (2008) and calculated in SPADE (Chao and Shen 2010). Significant dissimilarity was determined at a set value equal or above 0.15 (Jost 2008). Maximum parsimony-based haplotype networks were estimated using TCS version 1.21 (Clement et al. 2000). Mantel Tests were conducted using XLSTAT (Addinsoft 2010) to correlate dissimilarity values with differences in dissolved oxygen values between locations.

## Results

### Creatine kinase

**Figure 2:**
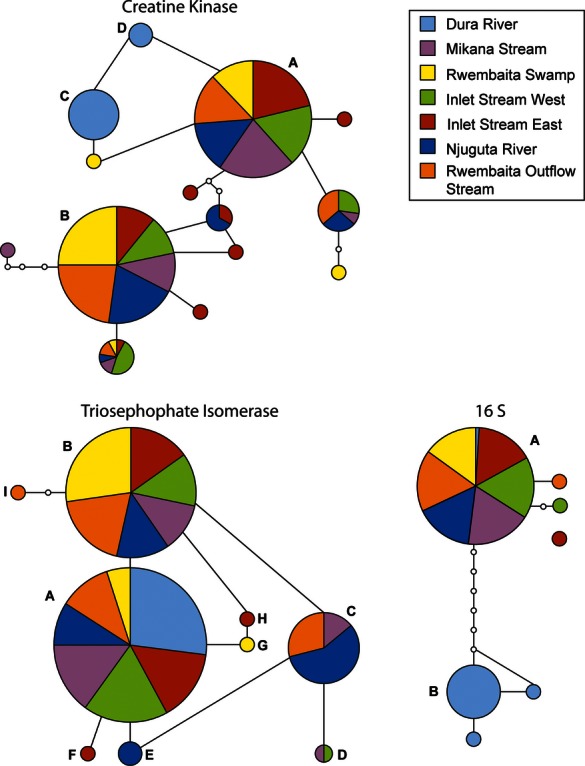
Parsimony network of haplotypes for the *Creatine Kinase* intron (CK), the *Triosephosphate isomerase* Intron (TPI) and the 16S gene. Coloured circles represent different haplotypes. Size of circles represent the number of individuals with a specific haplotype. Hollow nodes represent a single nucleotide polymorphism (SNP). Significance for connections is 0.95.

We amplified and sequenced 198 bp of the sixth intron of the CK-A gene and identified 14 haplotypes across the seven collection locations (Fig. 2; Table 1) with an overall haplotype diversity of 0.791. (Table 1). The Njuguta River location had the highest diversity (*Hd* = 0.766), while the Dura River had the lowest diversity (*Hd* = 0.189). The maximum number of mutational steps between any of the haplotypes was four, although this maximum was only observed between a single individual from Mikana Stream and the haplotype B. The most common haplotype for this intron was haplotype A, found in 42 of 135 individuals (31.11%, Fig. 2). This haplotype was observed in multiple individuals from all populations except the Dura River. In fact, all individuals from the Dura River site presented 1 of 2 unique haplotypes, C or D, not observed in any other population.

**Table 1:** Sampling locations and genetic diversity indices for CK and TPI introns as well as 16S gene

	Populations							
	
	Dura	ISE	ISW	Mik	NR	RS†	Rwe†	Overall
CK								
*N*_Seq_	20	19	20	20	19	20	17	135
Poly. Sites	1	7	3	7	4	3	6	11
*N*_Hap_	2	8	4	5	5	4	5	14
*Hd*	0.189	0.754	0.763	0.695	0.766	0.737	0.662	0.780
K	0.189	1.801	1.574	1.721	1.602	1.495	1.603	1.748
TPI								
*N*_Seq_	18	20	20	18	20	20	18	134
Poly. Sites	0	3	3	3	2	5	2	8
*N*_Hap_	1	4	3	4	4	4	3	9
*Hd*	0.000	0.616	0.542	0.607	0.763	0.647	0.386	0.608
K	0.000	0.721	0.705	0.843	1.000	0.968	0.477	0.785
16S								
*N*_Seq_	20	19	20	20	18	20	17	134
Poly. Sites		16	2	0	0	1	0	7
*N*_Hap_	4	2	2	1	1	2	1	4
*Hd*	0.284	0.105	0.100	0.000	0.000	0.100	0.000	0.284
K	0.895	1.684	0.200	0.000	0.000	0.100	0.000	0.895

Env. Type, Type of environment; *N*_Seq_, Number of individuals sequenced; Poly. sites, Polymorphic sites; *N*_Hap_, Number of haplotypes; *Hd*, Haplotype diversity; K, Average number of nucleotide differences; Dura, Dura River; ISE, Inlet Stream East; ISW, Inlet Stream West; Mik, Mikana Stream; NR, Njuguta River; RS, Rwembaita Outflow Stream; Rwe, Rwembaita Swamp.

† All sites are normoxic except RS and Rwe, which are hypoxic.

*G*_ST_ and Jost's *D* analyses suggest significant genetic differences between some of the populations at this locus (Table 2). *F*_ST_-values did not indicate significant divergence, but reflected and never contradicted *G*_ST_-values (data not shown). This pattern of *F*_ST_ showing similar, but not significant, patterns of differentiation to those in the *G*_ST_ analysis is consistent across all three loci and we have therefore presented only the *G*_ST_ values. Values for Jost's *D* dissimilarity ranged from −0.112 to 1.000, indicating variability between the Dura River population and all other populations (Table 2). Furthermore, Jost's *D* values indicated that the Rwembaita Swamp population and the Inlet Stream West population were genetically different from each other with a dissimilarity value of 0.192.

**Table 2:** *G*_ST_ and Jost's *D* values for the CK intron

Population	Dura	ISE	ISW	Mik	NR	RS	Rwe
Dura River		0.341*	0.339*	0.367*	0.343*	0.354*	0.376*
Inlet Stream East	1.000		0.011	−0.012	0.003	0.015	0.026
Inlet Stream West	1.000	0.073		0.004	0.008	0.003	0.041
Mikana Stream	1.000	−0.061	0.022		−0.010	−0.008	−0.001
Njuguta River	1.000	0.018	0.050	−0.054		−0.021	−0.003
Rwembaita Outflow Stream	1.000	0.090	0.020	−0.038	−0.122		−0.004
Rwembaita Swamp	1.000	0.092	0.192	−0.029	−0.017	−0.014	

*G*_ST_ values are above the diagonal, the dissimilarity matrix is below the diagonal. Dura, Dura River; ISE, Inlet Stream East; ISW, Inlet Stream West; Mik, Mikana Stream; NR, Njuguta River; RS, Rwembaita Outflow Stream; Rwe, Rwembaita Swamp.

* *P* < 0.05.

Overall, we found a significant positive relationship between geographical distance and both *G*_ST_ and the Jost's *D* dissimilarity coefficient with populations that were farther apart being the most genetically differentiated (*R*^2^ = 0.96, *P* < 0.0001 and *R*^2^ = 0.97, *P* < 0.0001 for *G*_ST_ and Jost's *D,* respectively). Due to the large distance between the Dura River population and all the other populations, additional analyses were conducted without the Dura River population. In the absence of the Dura River population, both *G*_ST_ and Jost's *D* were associated with the geographical distance in pairwise comparisons (*R*^2^ = 0.33; *P* < 0.0001 and *R*^2^ = 0.37; *P* < 0.0001 for *G*_ST_ and Jost's *D,* respectively). By contrast, we did not find a significant relationship between either genetic metric and dissolved oxygen at this locus, even when the Dura River was not included in the analysis.

### Triosephosphate isomerase

We amplified and sequenced 150 bp from the fourth intron of the TPI-A gene and identified nine different haplotypes across the seven populations (Fig. 2; Table 1) with an overall haplotype diversity of 0.608 (Table 1). Similar to the results from the CK locus, the Rwembaita/Njuguta Outflow Stream had the highest overall diversity (*Hd* = 0.763), while the Dura River was completely fixed at this locus with a haplotype diversity of 0.000. The maximum number of mutational steps between any of the nine haplotypes obtained was two. The most common haplotype for the TPI intron was haplotype B found in 66 of 134 individuals (49%, Fig. 2) and in all locations, suggesting a single source population for this haplotype. Haplotype B was also the only haplotype observed in the Dura river population. The next most common haplotype was A, found in 55 of 134 individuals (41%) and in all populations except the Dura River population.

Similar to CK, *G*_ST_ and Jost's *D* analyses suggest significant genetic difference at this locus between some of the populations (Table 3). Values for Jost's *D* dissimilarity ranged from −0.065 to 0.794 and also showed significant differences between the Dura River population and all the other populations as well as between the Rwembaita Swamp population and all other populations except with the Rwembaita/Njuguta Outflow Stream population (Table 3). Additionally, the Njuguta River population and the Inlet Stream West population were marginally dissimilar from each other (Jost's *D*-value = 0.129).

**Table 3:** *G*_ST_ and Jost's *D* values for the TPI intron

Population	Dura	ISE	ISW	Mik	NR	RS	Rwe
Dura River		0.228*	0.184*	0.188*	0.283*	0.323*	0.624*
Inlet Stream East	0.278		−0.017	−0.019	0.015	−0.005	0.094*
Inlet Stream West	0.177	−0.045		−0.025	0.033	0.017	0.149*
Mikana Stream	0.202	−0.049	−0.065		0.014	0.005	0.132*
Njuguta River	0.515	−0.066	0.129	0.064		−0.004	0.081*
Rwembaita Outflow Stream	0.482	−0.018	0.050	0.015	−0.018		0.033
Rwembaita Swamp	0.794	0.210	0.306	0.301	0.243	0.075	

*G*_ST_ values are above the diagonal, the dissimilarity matrix is below the diagonal. Dura, Dura River; ISE, Inlet Stream East; ISW, Inlet Stream West; Mik, Mikana Stream; NR, Njuguta River; RS, Rwembaita Outflow Stream; Rwe, Rwembaita Swamp.

* *P* < 0.05.

**Figure 3:**
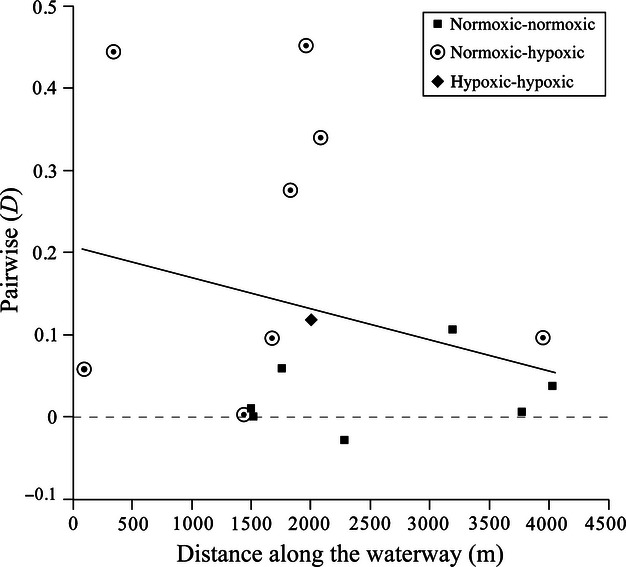
Relationship between Jost TPI intron *D* values as function of the geographical distance along the waterway. Black points with circles represent the comparison between hypoxic and normoxic sampling sites; black squares represent comparisons between normoxic-normoxic sampling sites; black diamonds represent comparisons between hypoxic and hypoxic sampling sites.

We observed a significant positive correlation between geographical distances and both genetic metrics (*R*^2^ = 0.38, *P* < 0.0001 and *R*^2^ = 0.46, *P* < 0.0001 for *G*_ST_ and Jost's *D,* respectively). Nevertheless, when the Dura River was removed from the analysis, these relationships disappeared (*P* = 0.31 and *P* = 0.35 for *G*_ST_ and Jost's *D,* respectively). By contrast, the relationships between the genetic metrics and dissolved oxygen between locations were significant when the Dura river was included (*R*^2^ = 0.25, *P* = 0.04 and *R*^2^ = 0.64, *P* < 0.0001 for *G*_ST_ and Jost's *D,* respectively), but stronger when this population was excluded from the analysis (*R*^2^ = 0.83, *P* < 0.0001 and *R*^2^ = 0.83, *P* < 0.0001 for *G*_ST_ and Jost's *D*, respectively; Fig. 3).

### 16S mitochondrial locus

We sequenced a 555 bp fragment of the 16S locus and identified seven haplotypes (Fig. 2) with an overall haplotype diversity of 0.287 (Table 1). In contrast to the results from the nuclear loci, in this mitochondrial data set, the Dura River location had the highest haplotype diversity (*Hd* = 0.284). The Mikana Stream, Njuguta River, and the Rwembaita Swamp all had only a single 16S haplotype and an *Hd* of 0.000. The most common 16S haplotype was haplotype A found in 112 of 134 individuals (83.6%). Haplotype B was observed in 17 of 134 individuals (12.7%) and only in individuals from the Dura River. The remaining haplotypes were found only in single individuals.

Similar to the two nuclear loci, *G*_ST_ and Jost's *D* analyses suggest significant genetic differences at this locus between some of the populations (Table 4). In particular, we observed a significant genetic isolation between individuals from the Dura River population and all other locations. Significant differences in *G*_ST_ and Jost's *D* were also observed between the Mikana River and the Njuguta River, the Mikana River and the Rwembaita Swamp, and the Njuguta River and Rwembaita Swamp (Table 4).

**Table 4:** *G*_ST_ and Jost's *D* values for the 16S gene

Population	Dura	ISE	ISW	Mik	NR	RS	Rwe
Dura River		0.658*	0.664*	0.740*	0.729*	0.664*	0.723*
Inlet Stream East	0.941		−0.013	0.000	0.000	−0.013	0.000
Inlet Stream West	0.941	−0.003		0.000	0.000	−0.013	0.000
Mikana River	0.942	0.003	0.000		1.000	0.000	1.000
Njuguta River	0.942	0.003	0.000	0.000		0.000	1.000
Rwembaita Outflow Stream	0.941	−0.003	−0.006	0.000	0.000		0.000
Rwembaita Swamp	0.942	0.003	0.000	0.000	0.000	0.000	

*G*_ST_ values are above the diagonal, the dissimilarity matrix is below the diagonal. Dura, Dura River; ISE, Inlet Stream East; ISW, Inlet Stream West; Mik, Mikana Stream; NR, Njuguta River; RS, Rwembaita Outflow Stream; Rwe, Rwembaita Swamp.

* *P* < 0.05.

Similar to the results from the nuclear loci, there was a positive and strong correlation between geographical and genetic distance in the 16S data set (*R*^2^ = 0.36, *P* = 0.08 and *R*^2^ = 0.94, *P* < 0.0001 for both *G*_ST_ and Jost's *D,* respectively). However, no correlation was observed between geographical distance and either *G*_ST_ (*P* = 0.363) or Jost's *D* (*P* = 0.834) when the Dura river was excluded from the analysis. Additionally, there was no correlation between the genetic metrics and dissolved oxygen even when the Dura River was removed from the analysis.

## Discussion

Results from this study indicate that both geographical distance and dissolved oxygen regime contribute to genetic isolation and population genetic structure in *B. neumayeri* in the Kibale National Park, Uganda. Based on both nuclear and mitochondrial loci analyses, the Dura River population is isolated from the other sampling locations by geographical distance and landscape features, and shows the highest degree of genetic isolation in comparison with all the other locations. When the Dura River location was removed from the analyses, however, the correlation between geographical distance and genetic differentiation disappears and a different pattern of differentiation is observed.

### Genetic structure of the *B. neumayeri* in the Kibale National Park

#### Distance vs. oxygen regimes

The relatively high level of genetic structure at the mitochondrial locus between the Dura River and all other locations indicates little if any gene flow between the two drainages. Furthermore, the high dissimilarity between the Dura River and all other sample locations observed at both nuclear introns suggests that the isolation of the Dura River is not a relatively recent event. The clustering of the Dura River population in the haplotype analyses further supports the idea that there is little to no effective migration occurring between the Dura River and the other locations. Two isolated groups are readily apparent in the 16S haplotype network: the Dura River population, and a second group including all the other populations. The number of fixed mutations at the 16S locus is unlikely to occur over a short period of isolation, suggesting that this isolation is not a seasonal event and one that has been established for many generations.

Recent physiological studies (Martínez et al. 2004, 2011) further support our hypothesis that genetic isolation of *B. neumayeri* is also associated with dissolved oxygen regime. Our results suggest reduced gene flow between populations from the two hypoxic Rwembaita locations and both the high oxygen locations and the normoxic Inlet Stream West populations, despite the fact that these two set of populations are only separated by 328 m (one of the shortest distances between locations). This isolation suggests that habitat-specific selection pressures may contribute to reduced gene flow (or alternatively, high gene flow may constrain local adaptation), as has been suggested in other studies (Hendry et al. 2002; Crispo 2008). The TPI intron data further suggest that genetic differentiation is occurring between individuals from the hypoxic Rwembaita Swamp and individuals from all the other sampling locations, except for individuals from the Rwembaita/Njuguta Outflow Stream, the only other hypoxic sampling location characterized by low-conductivity swamp water. Among all six sampling locations in the Rwembaita-Njuguta system, the Rwembaita/Njuguta Outflow Stream is the furthest from the Rwembaita Swamp (1997 m). In other words, the two hypoxic locations are genetically most similar while being geographically distant. The habitat in between these locations is a continuous papyrus swamp, possibly enabling swamp-dwelling fish to move more freely within the swamp system over broad distances. We observed strong correlations between DO and genetic dissimilarity at the TPI intron even when the Dura River is removed from the analysis, adding further support to our conclusion that oxygen regime is playing a significant role in determining genetic divergence and population structuring of *B. neumayeri* at micro-geographical scales.

Interestingly, studies of the widespread cichlid *P. multicolor* in the Mpanga River system provided no evidence of population genetic structure with respect to oxygen regimes; however, there was evidence for isolation by distance (Crispo and Chapman 2008). The different patterns of isolation seen in this study and the work from Crispo and Chapman (2008) may reflect differences in the species (i.e., cichlids vs. cyprinids), or differences in the level of resolution between the molecular loci (i.e., nuclear introns vs. microsatellites). Despite the fact that *P. multicolor* and *B. neumayeri* are from very phylogenetically distinct lineages, both occupy similar heterogeneous niches with respect to dissolved oxygen availability, and display similar patterns of divergence across DO gradients in morphological traits such as gill size. In *P. multicolor*, common-garden rearing studies have demonstrated a high degree of phenotypic plasticity in some traits related to oxygen uptake such as gill size, which may buffer the effects of local adaptation and allow for high rates of gene flow at the meta-population level (Chapman et al. 2008; Crispo 2008; Crispo and Chapman 2010). Further studies are needed to determine whether intron variation may exist across oxygen gradients in *P. multicolor*. While other environmental characters differ between low- and high-oxygen locations, several lines of evidence suggest that dissolved oxygen availability is driving divergence in physiological, behavioral, and morphological traits between *B. neumayeri* from low- and high-oxygen locations (Chapman and Liem 1995; Olowo and Chapman 1996; Chapman et al. 1999; Martínez et al. 2004, 2011). Our study therefore supports the hypothesis that non-geographical environmental factors can affect genetic variability between subpopulations in a continuous system (Peichel et al. 2001; Hendry et al. 2002; Lecomte and Dodson 2004; Bekkevold et al. 2005; Funk et al. 2005; McCairns and Bernatchez 2008). In the case of *B. neumayeri*, it is likely that divergent selection has led to variation in traits associated with oxygen uptake, in turn resulting in performance trade-offs for different respiratory phenotypes that contributes to the maintenance of divergence in both phenotype and genotype (Schaack and Chapman 2003). The role of phenotypic plasticity in contributing to oxygen-related trait divergence in *B. neumayeri* is an area for future study.

#### G_ST_ vs. Jost's *D*

This study also allowed us to compare and contrast the results from distinct metrics of genetic structure. Some limitations that have been pointed out for *G*_ST_ values (e.g., Charlesworth 1998; Nagylaki 1998; Jost 2008) are evident in our results. For example, in the analysis of the 16S data set, *G*_ST_-values revealed significant differences between *B. neumayeri* from the Rwembaita Swamp and both Mikana Stream and Njugata River; however, Jost's *D* showed no difference between these populations. It appeared that all but a single individual from the Dura River possessed a haplotype unique to this population, suggesting that *G*_ST_-values should approach 1 as opposed to 0.664–0.740 as indicated by our results. In comparison, Jost's *D*-values for the Dura River ranged from 0.941 to 0.942, a better indication of strong genetic differentiation. Our results, then, appear to be consistent with concerns raised in earlier studies (Charlesworth 1998; Nagylaki 1998; Jost 2008) that true genetic differentiation is not distinguishable when unique alleles exist in subpopulations, as well as when populations with different mean heterozygosities are compared. Similar differences were observed for the CK locus for which all the individuals of the Dura River presented one of four unique haplotypes indicating a very high differentiation (*G*_ST_-values = 0.339 to 0.374 and Jost's *D*-values = 1.000). Such discrepancies may cause misleading conclusions with respect to population structure leading to inappropriate management implications and we therefore advocate for a better investigation of the properties associated with different metrics used to estimate genetic differentiation. Furthermore, it is clear that *G*_ST_ may yield less realistic measure of population differentiation than Jost's *D* and caution must be taken when interpreting and comparing the results from different studies. Recently, Raeymaekers et al. (2012) empirically and theoretically compared the performance of *D* and *G*_ST_ in distinguishing population genetic structure and conclude that no single measure can quantify all aspects of genetic structure. The differences in resolution observed here between *D* and *G*_ST_ are consistent with this conclusion. Interestingly, both their empirical and theoretical work suggest that *D* is more useful in distinguishing longer term processes, and *G*_ST_ in recent demographic events, while our empirical results appear to point in the opposite direction. This difference in conclusion suggests that either the separation of the *B. neumayeri* populations that we have studied is more complicated than we appreciate, or, that more work is necessary to completely understand the difference in performance of these two metrics.

## Conclusion

Our study detected significant population differentiation in the African cyprinid *B. neumayeri* across dissolved oxygen regimes. We also observe strong genetic differentiation of the Dura River location from all other sites, likely a function of the large swimming distance and the presence of geographical barriers between the Dura River location and all of the other samples sites. The remaining six sampling locations, although geographically close to each other, present levels of genetic divergence associated with dissolved oxygen regime. Earlier studies on the biochemistry of this system found significant differences between different oxygen regimes (Martínez et al. 2004, 2011) and demonstrated that these differences were not plastic, conclusions most easily explained by some degree of genetic isolation between populations. We find that within the Rwembaita Swamp system, the two hypoxic locations are genetically similar to each other, while the four normoxic locations are genetically similar to each other. Combined with the earlier biochemical studies, our results support the idea that aquatic dissolved oxygen can act as a selective force limiting gene flow among populations of aquatic species and therefore should be considered when implementing conservation plans and be of future concern when assessing environmental impact of human activities, especially on sensitive ecosystems such as tropical watersheds.

## Data accessibility

DNA sequences have been submitted to GenBank.
